# The α-RECIST (RECIST 1.1 Combined With Alpha Fetoprotein): A Novel Tool for Identifying Tumor Response of Conversion-Radiotherapy for Unresectable Hepatocellular Carcinoma Before Hepatectomy

**DOI:** 10.3389/fonc.2022.905260

**Published:** 2022-05-24

**Authors:** Ying Xu, Yi Yang, Lu Li, Feng Ye, Xinming Zhao

**Affiliations:** ^1^ Department of Diagnostic Radiology, National Cancer Center/National Clinical Research Center for Cancer/Cancer Hospital, Chinese Academy of Medical Sciences and Peking Union Medical College, Beijing, China; ^2^ Department of Hepatobiliary Surgery, National Cancer Center/National Clinical Research Center for Cancer/Cancer Hospital, Chinese Academy of Medical Sciences and Peking Union Medical College, Beijing, China; ^3^ Key Laboratory of Gene Editing Screening and Research and Development (R&D) of Digestive System Tumor Drugs, Chinese Academy of Medical Sciences and Peking Union Medical College, Beijing, China

**Keywords:** hepatocellular carcinoma, radiotherapy, hepatectomy, tumor response, RECIST 1.1

## Abstract

**Purpose:**

To develop a novel criterion based on the response evaluation criteria in solid tumors (RECIST) 1.1 and alpha fetoprotein (AFP) and evaluate its performance in tumor response for patients with unresectable hepatocellular carcinoma (uHCC) receiving conversion-radiotherapy before hepatectomy.

**Method:**

From June 2012 to December 2020, a total of 39 patients with uHCC, who received intensity-modulated radiotherapy (IMRT) before hepatectomy, were retrospectively included in this study. Pre- and post-treatment contrast-enhanced magnetic resonance imaging (CE-MRI) scans were performed in all patients. Eight modified criteria were developed with the combination of RECIST 1.1, modified RECIST (mRECIST), and the percentage change of AFP, baseline AFP. The endpoint events were recurrence-free survival (RFS).

**Results:**

The median RFS and OS was 26.5 (IQR, 15.7-43.1), 38.8 (IQR, 18.4-53.6) months. An optimal revised evaluation criterion named α-RECIST (alpha fetoprotein-RECIST 1.1) was developed by combining the RECIST 1.1 with the AFP_Δ_ (cut-off value, 76%). Patients defined as responders by α-RECIST showed significantly better RFS and OS than those defined as non-responders (*p* = 0.035, 0.048). The other criteria (RECIST 1.1, mRECIST, α_Δ_-mRECIST, α_&Δ_-RECIST, α_&Δ_-mRECIST, α_BL_-RECIST, α_BL_-mRECIST, α_&BL_-RECIST, α_&BL_-mRECIST) all failed to identify responders from non-responders (*p* = 0.405, 0.201, 0.773, 0.424, 0.266, 0.060, 0.721, 0.644, 0.910, respectively) when correlated with RFS. Responders according to α-RECIST showed significant better RFS compared to non-responders [HR, 0.31 (95% CI: 0.10, 0.98); *p*=0.046], but no statistical significance was observed in terms of OS [HR, 0.33 (95% CI: 0.11, 1.05); *p* = 0.06].

**Conclusions:**

Patients identified as responders by α-RECIST provided significant better RFS. The α-RECIST criteria might be a promising tool for identifying tumor response of conversion-radiotherapy for unresectable hepatocellular carcinoma before hepatectomy.

## Highlights

1. Conventional RECIST 1.1, mRECIST failed to correlate responders over non-responders with survival benefit for uHCC patients receiving conversion-radiotherapy before hepatectomy.
2. The novel criterion (a-RECIST) is based on RECIST 1.1 combining with the percentage change of AFP (AFP_Δ_) for identifying tumor response of conversion-radiotherapy for uHCC patients before hepatectomy.
3. Patients identified as responders by α-RECIST provided significant better RFS. The α-RECIST criteria might be a promising tool for identifying tumor response of conversion-radiotherapy for uHCC patients before hepatectomy.

## Introduction

Liver cancer is the sixth most common cancer and the fourth leading cause of cancer-related deaths worldwide (1), in which hepatocellular carcinoma (HCC) is the most common histological subtype (2). Surgical therapy is the first-line curative therapeutic method for early-stage HCC (2). However, more than 70% of patients are diagnosed as unresectable HCC (uHCC) due to local vascular invasion or inadequate baseline hepatic function (3, 4). Conversion therapy, including regional and systemic therapies, originally aimed at palliation, has recently been reported to convert tumors from unresectable to resectable status and receive curative resection (5, 6), from which radiotherapy (RT) is considered a promising conversion therapy for uHCC with the development of radiotherapeutic technology (4, 7). Intensity-modulated radiotherapy (IMRT) can selectively deliver radiation to tumor regions with high precision and exquisitely conformal dose distribution (7–9).

Tumor response criteria for HCC mainly includes Response Evaluation Criteria in Solid Tumors version 1.1 (RECIST 1.1), modified Response Evaluation Criteria in Solid Tumors (mRECIST), and the European Association for the Study of Liver Diseases (EASL). RECIST 1.1 only considers tumor size and may underestimate therapeutic response (10). EASL and mRECIST may not be accurate for the identification of responders due to “pseudo-progression”, that is, persistent enhancement after radiotherapy did not necessarily indicate viable neoplasm (10–12). Currently, no clear-cut criterion has been established for tumor response of radiotherapy, especially conversion-radiotherapy (13). A reliable criterion for response evaluation of conversion-radiotherapy is crucial and urgent.

Besides the radiological characteristics, oncological characteristics such as serum α-fetoprotein (AFP) has also been reported as potential response indicators of HCC. A high level of serum AFP is an indicator of poor prognosis across all stages of HCC (14, 15). Decreased AFP level has been reported to correlate with prolonged progression free survival (PFS) and overall survival (OS) after systemic therapy (15, 16).

In this study, we aim to explore the optimal modified criteria based on radiological criteria (RECIST 1.1, mRECIST) and oncological characteristics (e.g., baseline AFP and AFP change) to assess tumor response for uHCC receiving conversion-radiotherapy before hepatectomy based on recurrence-free survival (RFS).

## Materials and Methods

### Patients

**Figure 1:**
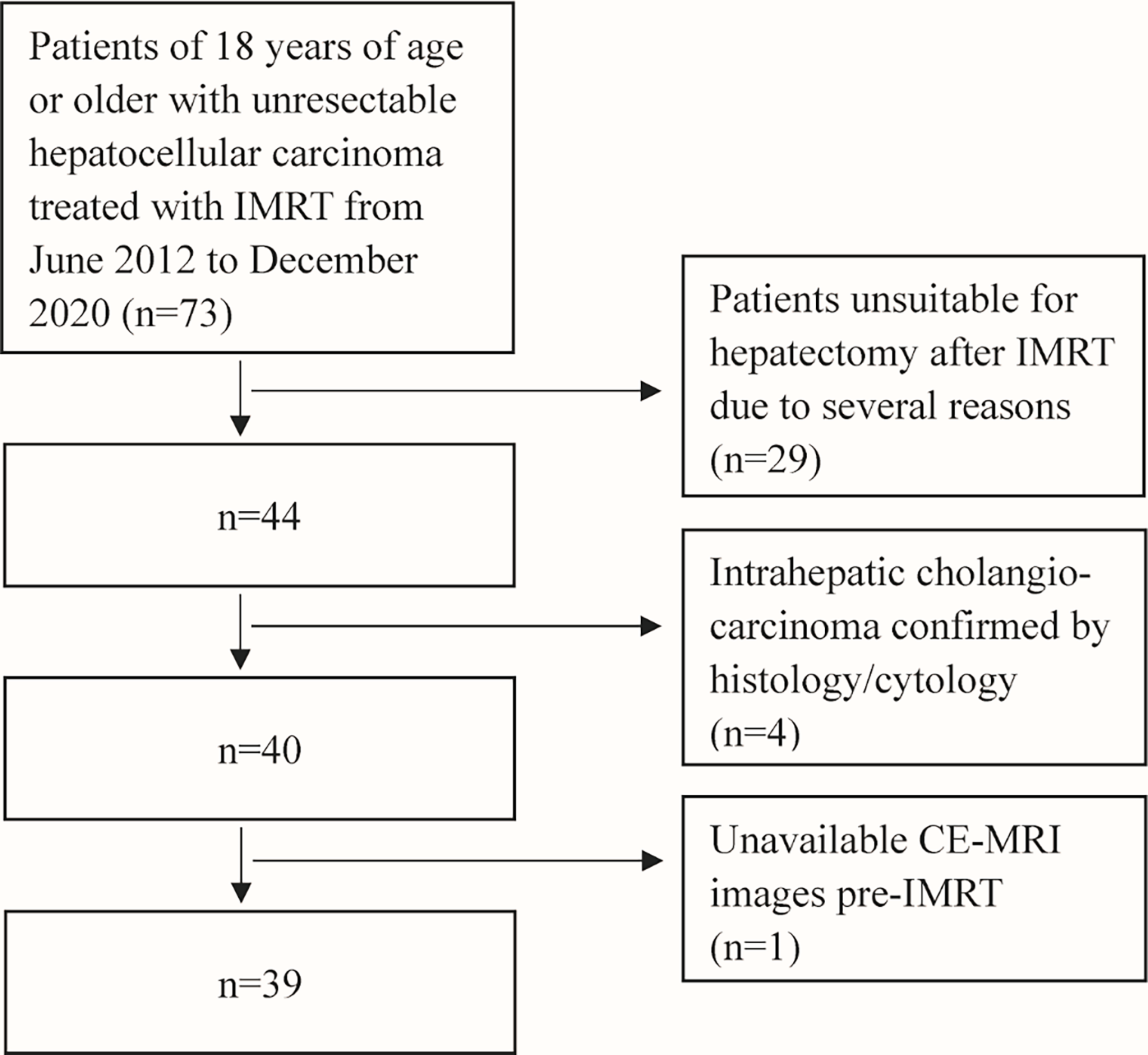
Flowchart of study population selection.

After searching the institutional medical database, sixty-nine patients with uHCC treated with IMRT from June 2012 to December 2020 were retrospectively included in this study. Inclusion criteria were: 1) patients with uHCC confirmed by liver biopsy; 2) patients without previous systemic therapy for HCC; 3) Eastern Cooperative Oncology Group Performance Status (ECOG PS) 0 or 1 and Child-Pugh score ≤6 points; 4) patients with both the contrast-enhanced magnetic resonance imaging (CE-MRI) of the abdomen within 1 month before IMRT as baseline and a follow-up CE-MRI scan after IMRT; 5) patients who had successful conversion-radiotherapy and subsequent hepatectomy. Exclusion criteria were: 1) patients unsuitable for hepatectomy after IMRT due to several reasons (such as inadequate hepatic functions); 2) baseline or follow-up CE-MRI scans were not within the predefined interval. A flowchart of the study population selection is shown in **Figure 1**. This study was approved by the Institutional Review Boards of National Cancer Center/Cancer Hospital and informed consent was waived for its retrospective design.

### IMRT

Preoperative IMRT was performed within 3 days after completion of all preoperative investigations and careful evaluation by the radiation clinicians. The gross tumor volume was defined as the tumor volume that was enhanced in the arterial phase on preoperative CE-MRI scans using the Pinnacle^3^ 9.0 treatment planning systems (Philips Healthcare, Andover, MA, USA). The clinical tumor volume (CTV) was drawn by adding 5–10 mm to the gross tumor volume. The planning target volume (PTV) was generated by adding 5mm in the anterior–posterior and left–right directions and 10mm in the cranial–caudal direction from the CTV considering respiratory liver motion and set-up errors. The prescription dose to 95% of the PTV was planned at 50-60 Gy in 25-30 fractions over 5-6 weeks. The final prescription dose was determined according to dose constraints for organs at risk.

### Hepatectomy

After completion of IMRT, the evaluation of liver function and MRI scans were performed before hepatectomy. Resection ranges were decided by clinical surgeons based on individual detailed status of each patient according to the tumor size, number, location, and relationship with the major vascular structures.

### Imaging Acquisition

MRI scans of the abdomen at baseline and follow-up of all patients were performed on three scanners: GE Discovery MR 750 3.0T, GE Pioneer 3.0T, and Siemens Prisma 3.0T. Routine liver MRI protocol included non-fat suppressed coronal single-shot fast/turbo spin-echo T2WI (SS-FSE/HASTE), axial fat suppressed fast/turbo spin echo (FSE/TSE) T2WI, T1WI in- and out-of- phase, DWI and dynamic CE-T1WI images. For dynamic CE-T1WI, unenhanced, early and late arterial phases (using fluoro trigger or carebolus technique), portal venous phase (60s), late venous phase (180s) were obtained using a 3D T1WI breath-hold fat-suppressed spoiled gradient-recall echo sequence (LAVA or VIBE) before and after intravenous administration of gadodiamide (0.5 mmol/ml, GE Healthcare) at a dose of 0.2 ml/kg body weight and an injection rate of 2ml/s. Respiratory-triggered or diaphragm-navigated DWI with an axial single-shot spin echo, echo-planar imaging (EPI) sequence with DW gradients (b value 0/50 and 800 s/mm^2^) was applied in three orthogonal directions (slice thickness/space: 6.0/1.0 mm, FOV: 34−38 cm, matrix size: 128×128, NEX: 4).

### Definitions of Modified Criteria

The baseline and follow-up AFP levels were calculated within one week before (AFP_BL_) and after (AFP_FU_) the IMRT. The percentage change from AFP_BL_ to the AFP_FU_ was calculated as the following formula: AFP_Δ_ = (AFP_FU_-AFP_BL_)/AFP_BL._ The optimal cutoff values of AFP_Δ_ and AFP_BL_ were determined according to RFS. Eight modified radiological criteria by combining RECIST, mRECIST with AFP_Δ_, AFP_BL_ were as follows, α_Δ_-RECIST, α_Δ_-mRECIST, α_&Δ_-RECIST, α_&Δ_-mRECIST, α_BL_-RECIST, α_BL_-mRECIST, α_&BL_-RECIST, α_&BL_-mRECIST **(** **Table 1** **)**. The optimal criterion would be selected as alpha fetoprotein-RECIST (α-RECIST) at last.

**Table 1:** Different tumor response evaluation criteria based on RECIST 1.1, mRECIST and AFP_BL_, AFP_Δ_.

	CR	PR	SD	PD
**RECIST 1.1**	Disappearance of all target lesions. No new lesions.	≥30% decrease in tumor size of target lesions. No new lesions.	Neither PR nor PD.	≥20% increase in tumor size of the target lesions, as well as an absolute increase of at least 5 mm. New lesions.
**mRECIST**	Disappearance of any intra-tumor arterial enhancement in all target lesions.	≥30% decrease in tumor size of the viable (enhancement in the arterial phase) target lesions, taking as reference the baseline target lesions.	Neither PR nor PD.	≥20% increase in tumor size of the viable (enhancing) target lesions, taking as reference the smallest sum of the diameters of target lesions since treatment started. New lesions.
**α_Δ_-RECIST**	–	≥76% decrease in AFP change **or** ≥30% decrease in tumor size of the target lesions. No new lesions.	–	–
**α_Δ_-mRECIST**	–	≥76% decrease in AFP change **or** ≥30% decrease in tumor size of the viable target lesions, taking as reference the baseline target lesions.	–	–
**α_&Δ_-RECIST**	–	≥76% decrease in AFP change **and** ≥30% decrease in tumor size of the target lesions. No new lesions.	–	–
**α_&Δ_-mRECIST**	–	≥76% decrease in AFP change **and** ≥30% decrease in tumor size of the viable target lesions, taking as reference the baseline target lesions.	–	–
**α_BL_-RECIST**	–	AFP_BL_≥54.4ng/ml **or** ≥30% decrease in tumor size of the target lesions. No new lesions.	–	–
**α_BL_-mRECIST**	–	AFP_BL_≥54.4 ng/ml **or** ≥30% decrease in tumor size of the viable target lesions, taking as reference the baseline target lesions.	–	–
**α_&BL_-RECIST**	–	AFP_BL_≥54.4 ng/ml **and** ≥30% decrease in tumor size of the target lesions. No new lesions.	–	–
**α_&BL_-mRECIST**	–	AFP_BL_≥54.4 ng/ml **and** ≥30% decrease in tumor size of the viable target lesions, taking as reference the baseline target lesions.	–	–

CR, complete response; PR, partial response; SD, stable disease; PD, progressive disease.

### Image Analysis and Radiological Evaluation

Baseline and follow-up CE-MRI images were analyzed on Advantage Workstation (GE Medical Systems, USA). On baseline CE-MRI images, target lesions should be at least 1.0 cm for the longest diameter according to RECIST 1.1. For each patient, a maximum of two lesions per organ and five lesions in total were selected (17). The arterial phase was chosen to measure the sum of the longest diameter (SLD) of target lesions and the SLD of viable (enhancing) target lesions due to the fact that HCC is abundant with blood supply and shows obvious enhancement in the arterial phase (18–20). We calculated the relative changes of the SLD and the SLD of viable (enhancing) areas of all target lesions from baseline to the first follow-up CE-MRI evaluation for each patient, respectively. Non-target lesions were also evaluated according to RECIST 1.1 and mRECIST and the final evaluation results were based on both the target lesions and non-target lesions. Complete response (CR), partial response (PR), stable disease (SD), and progressive disease (PD) were determined by RECIST1.1, mRECIST and 8 modified radiological criteria. Responders were defined as patients with CR or PR, while non-responders were defined as patients with SD or PD.

Radiological evaluation was performed by two radiologists (Radiologist 1: L.L. M.D with 10 years of experience in abdominal radiology; Radiologist 2: Y.X. M.D with 5 years of experience in abdominal radiology) on baseline and the first follow-up CE-MRI scans. The discrepancy between the two radiologists was adjudicated by a third senior radiologist (Radiologist 3: F.Y. M.D with 18-years of experience in abdominal radiology) to reach a consensus among the three radiologists. All the three radiologists were blinded to the patients’ clinical data and outcome.

### Pathologic Response Evaluation

The per-lesion-based matching between each lesion on MRI and pathologic specimens was performed by two pathologists and a third pathologist was assigned to reach a consensus among the three pathologists when there was discrepancy. All the three pathologists were blinded to the imaging response results. A minimum of one slide per centimeter of each tumor was evaluated. The percentage of viable tumor region relative to the total tumor region was determined by the pathologists based on hematoxylin and eosin staining slides. Accordingly, the target lesions were categorized as pathologic complete response (no residual cancer cells), pathologic major response (1% to 49% residual cancer cells), and minor response (≥50% residual cancer cells) (21).

### Endpoint of Study

The primary endpoint of this study was RFS, which was calculated from the date of radical hepatectomy to recurrence or the last follow-up. The secondary endpoint was OS which was defined as the time from hepatectomy until death by any cause or the last follow-up. For survival analyses, the last follow-up was completed on August 7^th^, 2021. Recurrence included intrahepatic metastases assessed using MRI of the upper abdomen and extrahepatic metastases detected using chest-abdomen-pelvis CT following hepatectomy.

### Statistical Analysis

The RFS and OS curves of different criteria were prepared using the Kaplan-Meier method with the log-rank test. Univariate Cox regression analysis was performed to calculate the hazard ratios of responders to non-responders for RECIST 1.1 and α-RECIST criteria. The relationship of α-RECIST criteria and pathologic response were evaluated according to the Spearman’s correlation analysis. All the above statistical analyses were performed by SPSS 22.0 (SPSS Inc., Chicago, IL, USA). The Waterfall plots and the histogram were conducted with GraphPad Prism v.6 (GraphPad Software, La Jolla, CA, USA). X-tile 3.6.1 software (22) (Yale University, New Haven, CT, USA) was used to determine the optimal cut-off values for AFP_Δ_ and AFP_BL_ based on RFS. Duration of follow-up was calculated by the reverse Kaplan-Meier estimate of RFS and OS (23). Weighted k statistics were used to evaluate the inter-reader variability of tumor response between two radiologists, respectively. The weighted k coefficients were stratified as follows: 0.81-1.00, almost perfect; 0.61-0.80, substantial; 0.41-0.60, moderate; 0.21-0.40, fair; ≤0.20, poor (24). A two-tailed *p* value <0.05 was considered statistically significant.

## Results

### Baseline Characteristics

The percentage rate of successful conversion-radiotherapy was 58.0% (39/69). Thirty-nine patients with 41 target lesions were finally included in this study. The median duration of IMRT was 1.1 months [interquartile range (IQR), 1.0-1.2]. The median time from IMRT completion to the first response evaluation was 1.1 months (IQR 0.9-1.3). The median time from IMRT completion to hepatectomy was 2.3 months (IQR 1.7-3.4). More baseline characteristics of patients included were shown in **Table 2**.

**Table 2:** Patient and baseline characteristics.

No. of patients	n = 39
Age, years (mean ± SD)	53.0 ± 10.6
Male sex (%)	36(92.3)
ECOG PS	
0	16 (41.0)
1	23 (59.0)
Macrovascular invasion	
Yes	13 (33.3)
No	26 (69.7)
Microvascular invasion	
Yes	9 (23.1)
No	19 (48.7)
NA	11 (28.2)
Extrahepatic disease	
Yes	3 (7.7)
No	36 (92.3)
Reason for unresectable	
Macrovascular invasion	27 (69.2)
Insufficient remnant liver function	11 (28.2)
Multiple lesions	1 (2.6)
Baseline α-fetoprotein≥200ng/ml	19 (48.7)
Child-Pugh class A	39 (100.0)
Liver cirrhosis (investigator assessed)	
Yes	33 (84.6)
No	6 (15.4)
Etiology of HCC: Hepatitis B virus	
Yes	34 (87.2)
No	5 (12.8)
Number of target lesions	
1	37 (94.9)
≥2	2 (5.1)
Tumor size (cm)	7.0 (4.9-9.5)
<5	11 (28.2)
5-10	19 (48.7)
>10	9 (23.1)
Tumor differentiation	
Type I/II	20 (51.3)
Type III/IV	16 (41.0)
NA	3 (7.7)
First-line therapy	39(100.0)
Duration of IMRT (months)	1.1 (1.0-1.2)
Time between IMRT completion and first evaluation(months)	1.1 (0.9-1.3)
Time between IMRT completion and hepatectomy (months)	2.3 (1.7-3.4)

ECOG PS, Eastern Cooperative Oncology Group Performance Status; NA, Not available.

Data are n (%) or median (IQR), unless otherwise specified.

### Tumor Response Evaluation

**Figure 2:**
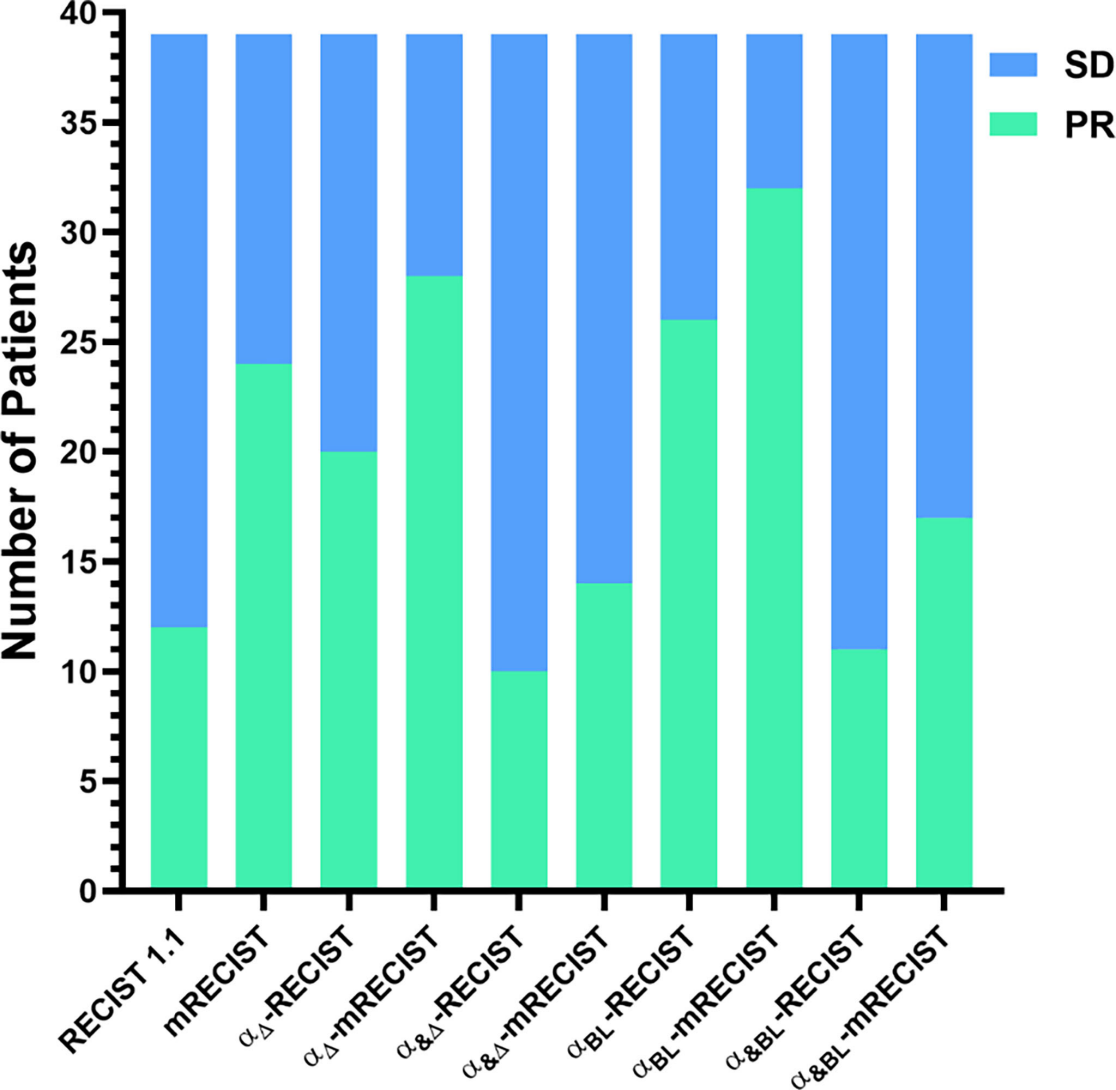
Comparisons of evaluation categories among the different criteria.

**Figure 3:**
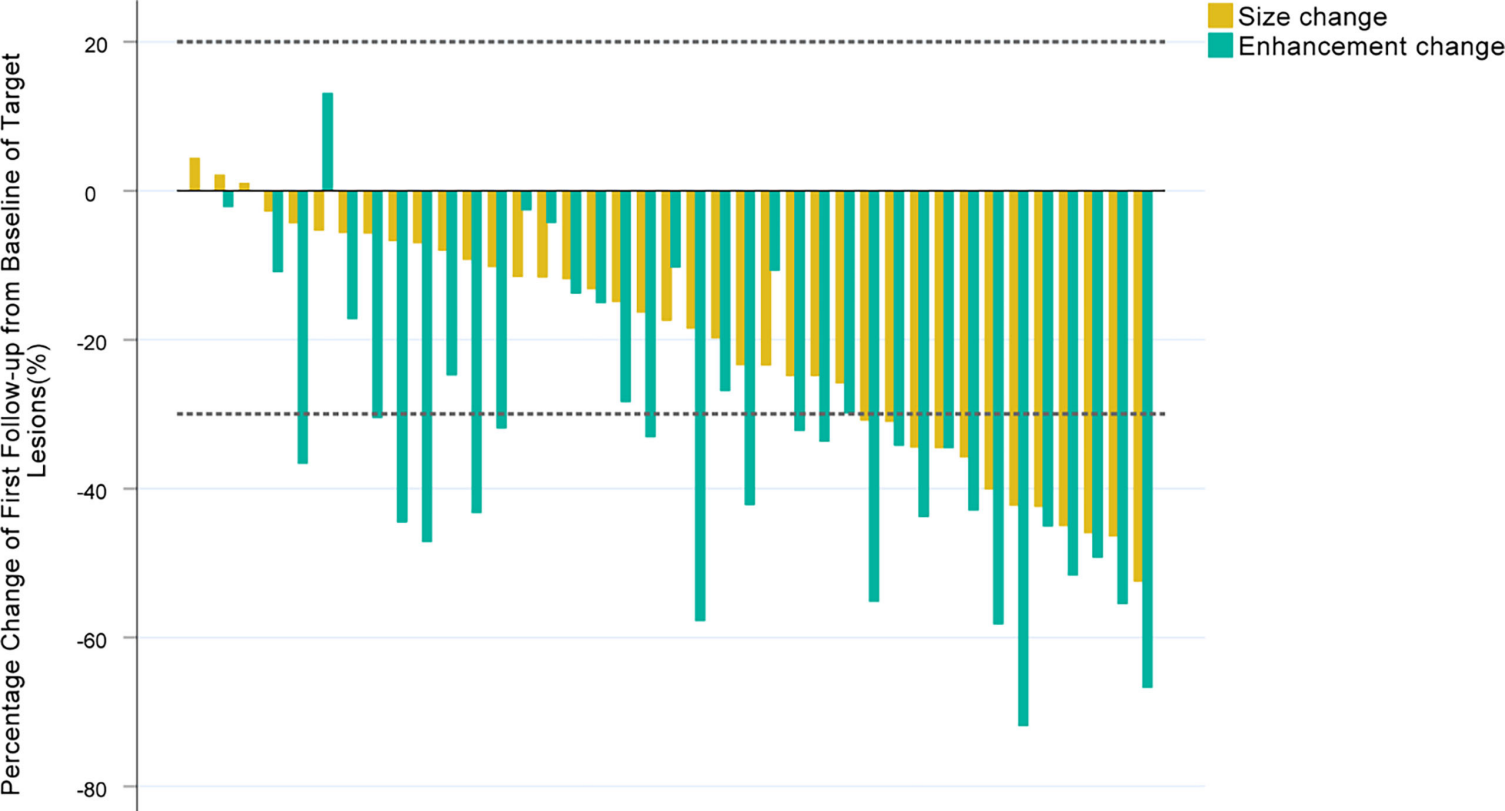
Waterfall plots summarizing the maximum percent change from baseline in the SLD, the SLD of viable (enhancing) areas of all the target lesions on the first follow-up CE-MRI scan after IMRT for each patient. The two adjacent bars in each group represent one patient. The dashed lines indicated size thresholds (+20%, -30%) for RECIST 1.1 and mRECIST criteria.

**Figure 4:**
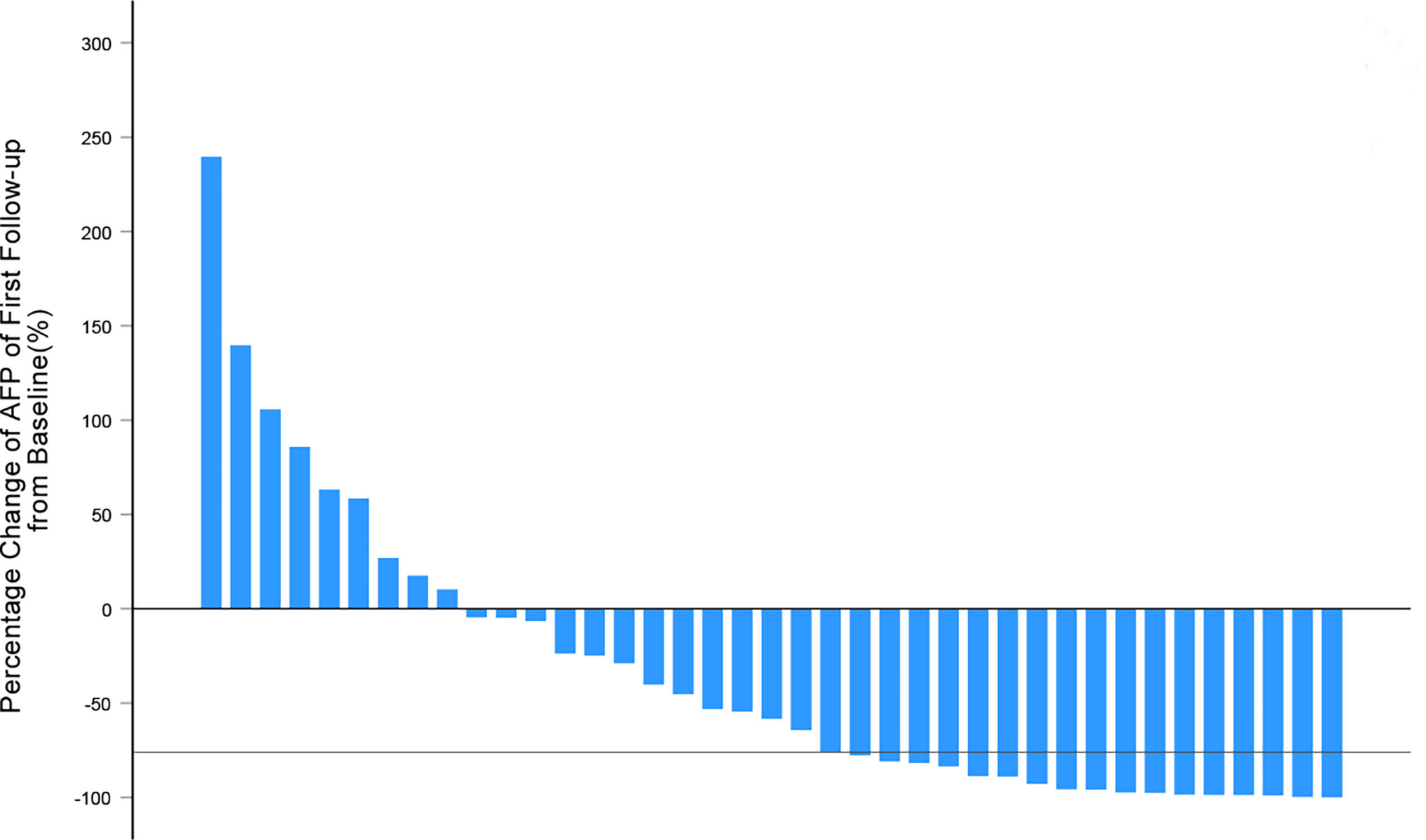
Waterfall plots summarizing the maximum percentage change of AFP from baseline on the first follow-up after IMRT for each patient. The solid line indicated AFP change thresholds (-76%) for α-RECIST criteria.

In the first tumor response evaluation, 12 (30.8%), 24 (61.5%), 20 (51.3%), 28 (71.8%), 10 (25.6%), 14 (35.9%), 26 (66.7%), 32 (82.1%), 11 (28.2%), 17 (43.6%) patients were diagnosed as PR and 27 (69.2%), 15 (38.5%), 19 (48.7%), 11 (28.2%), 29 (74.4%), 25 (64.1%), 13 (33.3%), 7 (17.9%), 28 (71.8%), 22 (56.4%) patients were diagnosed as SD according to RECIST 1.1, mRECIST, α_Δ_-RECIST, α_Δ_-mRECIST, α_&Δ_-RECIST, α_&Δ_-mRECIST, α_BL_-RECIST, α_BL_-mRECIST, α_&BL_-RECIST, α_&BL_-mRECIST, respectively. None of the patients were identified as CR or PD. The demonstration of evaluation categories among the different criteria are shown in **Figure 2**. For each patient, the changes of the SLD, the SLD of viable (enhancing) areas of all the target lesions on the first follow-up CE-MRI after IMRT are presented in **Figure 3** and the AFP_Δ_ is presented as **Figure 4**, respectively.

### Correlations of Different Criteria With RFS and OS

Seventeen (43.6%) patients occurred recurrence and 16 (41.0%) patients died during a median follow-up duration of 52.8 months (95% CI 43.8-61.8 months). The median RFS and OS time was 26.5 (IQR, 15.7-43.1 months), 38.8 months (IQR, 18.4-53.6 months). The 1-year, 2-year and 3-year RFS rates were 82.1%, 70.6%, 59.4%, respectively.

**Figure 5:**
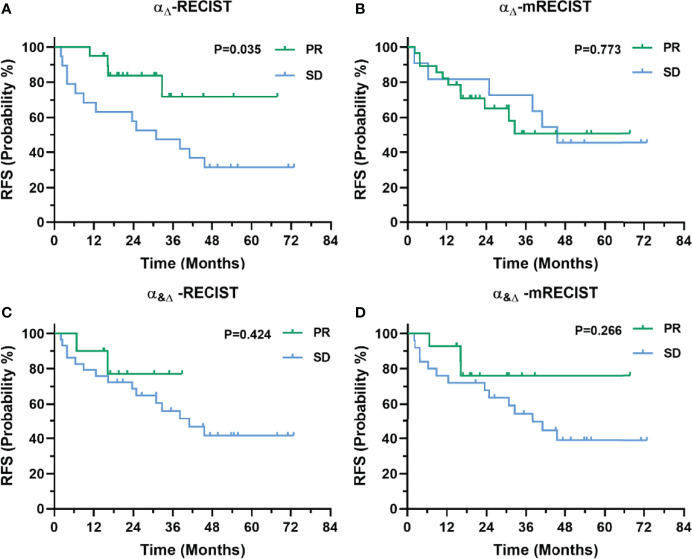
Kaplan-Meier curves for RFS of 39 patients with uHCC undergoing IMRT before hepatectomy as categorized by the α_Δ_-RECIST **(A)**, α_Δ_-mRECIST **(B)**, α_&Δ_-RECIST **(C)**, and α_&Δ_-mRECIST **(D)** criteria.

In terms of RFS, responders according to RECIST 1.1 and mRECIST showed no significant difference compared with non-responders (*p* = 0.405 and 0.201, respectively, **Supplemental Materials Figures 1A, B**). Patients with no less than 76% decrease in AFP_Δ_ showed significantly better RFS than those who did not (*p* = 0.042, **Supplemental Materials Figure 1C**). Patients with no less than 54.4ng/ml in AFP_BL_ showed a trend with better RFS than those who did not (*p* = 0.138, **Supplemental Materials Figure 1D**). Patients defined as PR by α_Δ_-RECIST showed significantly better RFS than those defined as SD (*p* = 0.035, **Figure 5A**). Responders according to the other criteria (α_Δ_-mRECIST, α_&Δ_-RECIST, α_&Δ_-mRECIST, α_BL_-RECIST, α_BL_-mRECIST, α_&BL_-RECIST, and α_&BL_-mRECIST) showed no significant difference in terms of RFS compared with non-responders (*p* = 0.773, 0.424, 0.266, 0.060, 0.721, 0.644, 0.910, **Figures 5B–D** and **Supplemental Materials Figures 2A–D**, respectively).

In terms of OS, responders according to RECIST 1.1 and mRECIST showed no significant difference compared with non-responders (*p* = 0.508, 0.103, **Supplemental Materials Figures 3A, B**). Patients defined as PR by α_Δ_-RECIST showed significantly better OS than those defined as SD (*p* = 0.048, **Supplemental Materials Figure 3C**). Patients with no less than a 76% decrease in AFP_Δ_ showed a trend with better OS than those who did not, though statistical significance was not detected (*p* = 0.180, **Supplemental Materials Figure 3D**).

### Correlations of Pathologic Response With RFS

Of the 39 patients receiving hepatectomy, 15 and 24 patients had pathologic major and minor response, respectively. None of the patients had pathologic complete response. Patients with pathologic major response showed no significant difference in terms of RFS compared with those with minor response (*p* = 0.798, **Supplemental Materials Figure 4**).

### Correlations of α-RECIST Evaluation With Pathologic Response

**Figure 6:**
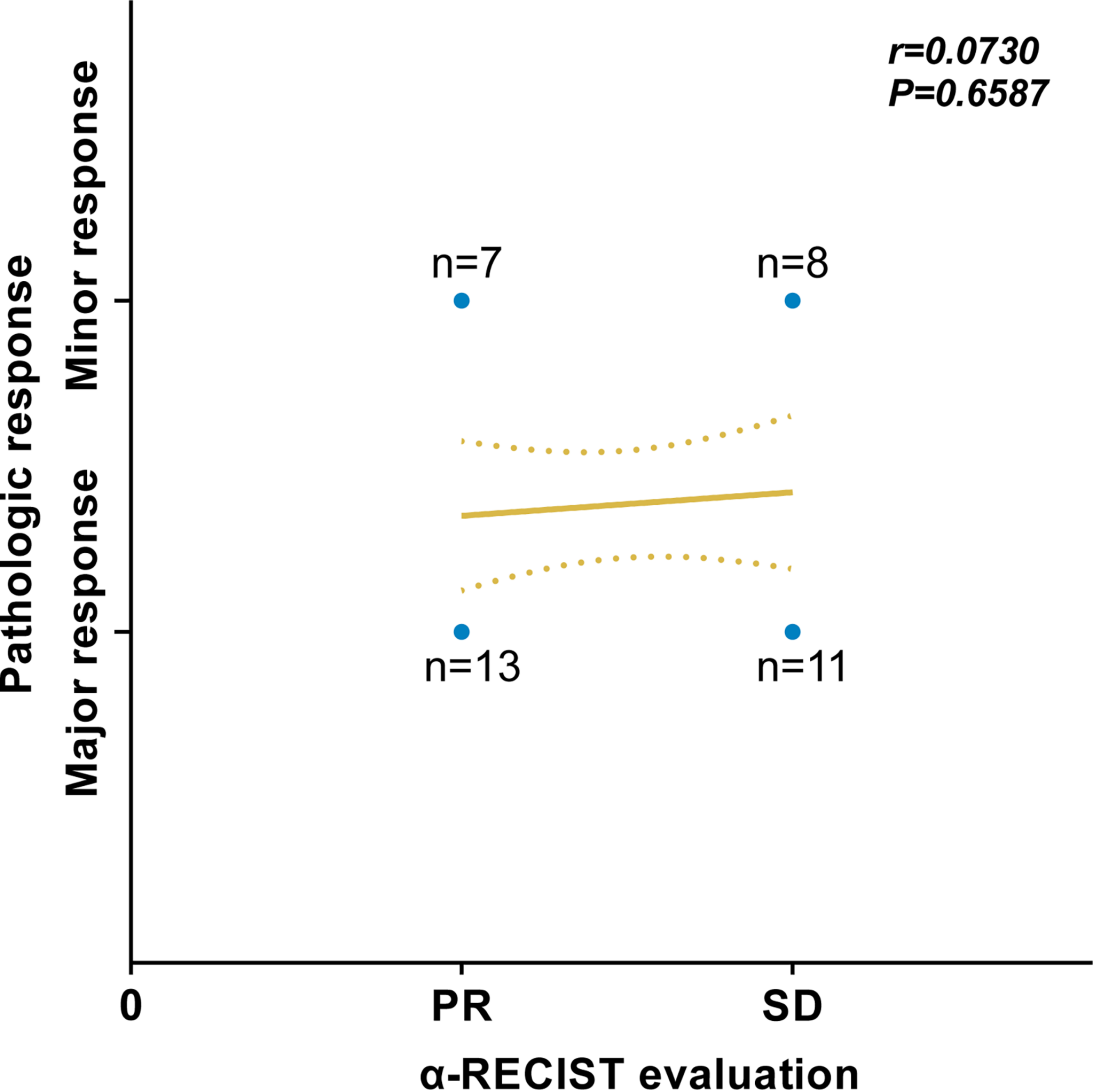
Correlation between α-RECIST evaluation and pathologic response evaluation.

Of all 20 patients defined as PR by α-RECIST, 13 and 7 patients had pathologic major and minor response, respectively. Nineteen patients were defined as SD by α-RECIST, 11 and 8 patients had pathologic major and minor response, respectively. According to the Spearman’s correlation analysis, no significant correlation was detected between α-RECIST evaluation and pathologic response evaluation (r = 0.0730, 95% CI: -0.2573 to 0.3880, *P* = 0.6587, **Figure 6**).

### The Superiority of α-RECIST Criteria

**Figure 7:**
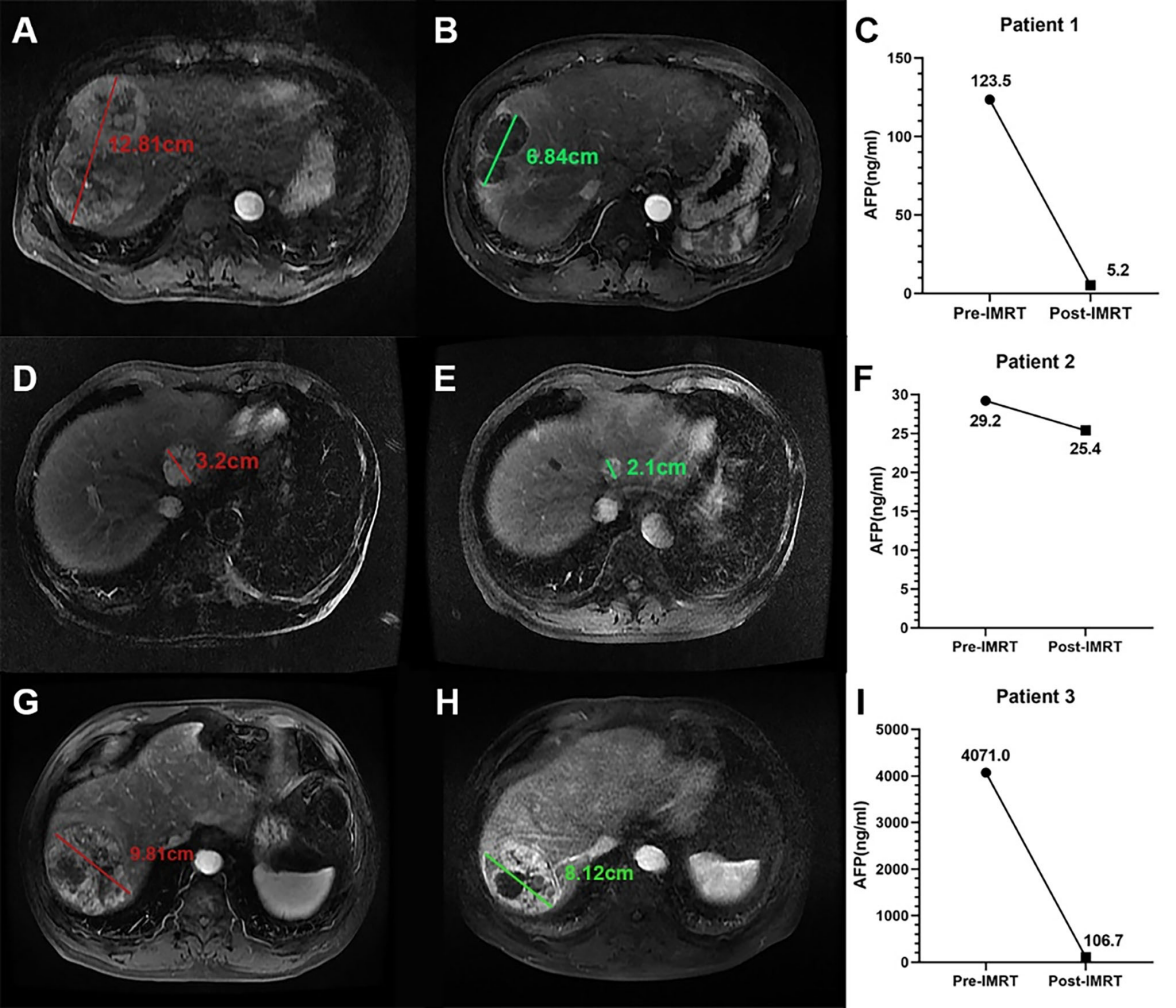
Three example cases of PR defined by the α-RECIST. **(A, B)** present the SLD on arterial phase images of pre- and post-IMRT CE-MRI scans for patient 1, and the size change is -42.0%; **(C)** presents the serum AFP levels before and after IMRT for patient 1, and the AFP_Δ_ is -95.8%. **(D, E)** present the SLD on arterial phase images of pre- and post-IMRT CE-MRI scans for patient 2, and the size change is -34.0%; **(F)** presents the serum AFP levels before and after IMRT for patient 2, and the AFP_Δ_ is -15.0%. **(G, H)** present the SLD on arterial phase images of pre- and post-IMRT CE-MRI scans for patient 3, and the size change is -17.0%; **(I)** presents the serum AFP levels before and after IMRT for patient 3, and the AFP_Δ_ is -97.4%. For patient 1, both the size decrease and the AFP decrease degree exceed the threshold values; For patient 2, only the size decrease degree exceed the threshold value; For patient 3, only the AFP decrease degree exceed the threshold value. All the three patients are defined as PR by α-RECIST.

Above all, α_Δ_-RECIST could enable identification of responders and significantly correlate with both RFS and OS. As a result, α_Δ_-RECIST was defined as the α-RECIST criteria. As is shown in **Figure 7**, α-RECIST criteria could identify three patterns of PR: fulfilling both PR defined by RECIST 1.1 and by changes of AFP, by fulfilling PR defined by RECIST 1.1 alone, and by fulfilling changes of AFP alone. Responders, according to α-RECIST, showed significantly better RFS compared to non-responders [HR, 0.31 (95% CI: 0.10, 0.98); *p* = 0.046] and no statistical significance was observed in terms of OS [HR, 0.33 (95% CI: 0.11, 1.05); *p* = 0.06] (**Table 3** **)**. RECIST 1.1 failed to correlate responders with survival benefit [RFS: HR, 0.59 (95% CI: 0.17, 2.11), *p* = 0.419; OS: HR, 0.65 (95% CI: 0.18, 2.33), *p* = 0.512]. The cumulative 1-year, 2-year, and 3-year RFS rates were 95.0%, 83.8%, 71.8%, respectively, in responders and 68.4%, 57.9%, 47.4%, respectively, in non-responders by α-RECIST criteria (*p* = 0.035). The cumulative 1-year, 2-year, and 3-year OS rates were 100.0%, 94.4%, 80.4%, respectively, in responders and 78.9%, 63.2%, 57.9%, respectively, in non-responders by α-RECIST criteria (*p*=0.048).

**Table 3:** Univariate hazard ratios for recurrence (RFS) and death (OS).

Parameter	RFS Hazard Ratios	*P* Value	OS Hazard Ratios	*P* Value
α-RECIST	0.31(0.10, 0.98)	0.046	0.33 (0.11, 1.05)	0.06
RECIST 1.1	0.59 (0.17, 2.11)	0.419	0.65 (0.18, 2.33)	0.512

Data in parentheses are 95% CIs.

### Inter-Reader Variability

The inter-reader agreements for RECIST 1.1, mRECIST, and α-RECIST criteria were 94.9%, 97.4%, 100.0%, respectively. The weighted k coefficients were 0.89 (0.74-1.04), 0.95 (0.85-1.05), 1.00 (1.00-1.00), respectively, as shown in **Table 4**.

**Table 4:** Response rates according to evaluation criteria for two independent radiologists with inter-reader agreement (*n*=39).

Tumor response, n (%)	RECIST 1.1		mRECIST		α-RECIST	
	R1	R2	R1	R2	R1	R2
CR	0 (0)	0 (0)	0 (0)	0 (0)	0 (0)	0 (0)
PR	10 (25.6)	12 (30.8)	23 (59.0)	24 (61.5)	20 (51.3)	20 (51.3)
SD	29 (74.4)	27 (69.2)	16 (41.0)	15 (38.5)	19 (48.7)	19 (48.7)
PD	0 (0)	0 (0)	0 (0)	0 (0)	0 (0)	0 (0)
Inter-reader agreement, n (%)	37 (94.9)		38 (97.4)		39 (100.0)	
Weighted k (95% CI)	0.89 (0.74-1.04)		0.95 (0.85-1.05)		1.00 (1.00-1.00)	

R1, Radiologist 1; R2, Radiologist 2.

## Discussion

Currently, no clear-cut criterion has been established for tumor response of patients with uHCC treated with IMRT. In this study, we investigated different modified versions of tumor response criteria by combining AFP_Δ_, AFP_BL_ with RECIST 1.1, mRECIST. It was demonstrated that responders identified by α-RECIST were associated with significant longer RFS (HR, 0.31 [95% CI: 0.10, 0.98]; *p* = 0.046), superior than the other evaluation criteria including RECIST 1.1, mRECIST, and pathologic response evaluation. The cumulative 1-year, 2-year, and 3-year RFS rates were 95.0%, 83.8%, 71.8%, respectively, in responders and 68.4%, 57.9%, 47.4%, respectively, in non-responders by α-RECIST criteria.

The most widely used criteria for tumor response in patients with HCC was RECIST 1.1 (4). In our study, responders detected by RECIST 1.1 showed a trend for longer RFS than non-responders, while no statistical significance was reached (*p* = 0.405). Since proposed in 2009 (25), mRECIST has been widely utilized in response evaluation of HCC after various treatment, including chemotherapy, targeted therapy, and immunotherapy (26–28). Currently, mRECIST has also been used in several clinical trials for tumor response in HCC treated with radiation (29–31). In our study, mRECIST failed to identify responders from non-responders with RFS benefit (*p* = 0.201). Park et al. (11) demonstrated a proportion of 46% patients showed arterial hypervascularity in parenchyma surrounding the original tumor at 3 months post radiotherapy, which was termed as “pseudo-progression” which probably resulted from subtotal collagenous occlusion of small hepatic vein branches and subsequent impaired outflow of blood and hyperemia (32). Thus, the persistent enhancement after radiotherapy did not necessarily indicate viable neoplasm, which could explain the limited value of mRECIST in tumor response for patients with uHCC treated with IMRT.

RECIST “or” changes of AFP gave the best separation of curves, while RECIST and changes of AFP were not predictive of RFS. We think it may be explained by the following reasons based on unidimensional diameter, RECIST 1.1 may underestimate tumor response and has been reported to have poor correlations with clinical outcomes in patients with HCC after systemic therapies (19, 33). After reviewing many radiological tumor response evaluations in clinical trials of our institution, tumor response usually occurs with minimal size shrinkage, which is usually insufficient to meet RECIST-defined response threshold (30%); meanwhile, AFP decrease quickly and distinctly after radiotherapy. As a result, some responders with minimal size shrinkage will be detected by the α-RECIST (RECIST or changes of AFP). As for RECIST 1.1 “and” changes of AFP, it seems too strict to define PR and it reclassified 10 α-RECIST-defined responders as non-responders. As a result, it may not detect the early response of responders with minimal size shrinkage. We noticed the insensitivity of the unidimensional diameter by RECIST 1.1 and tried to establish a novel criterion combined with the biochemical index AFP. The α-RECIST criteria reclassified eight RECIST-defined non-responders as responders and did not reclassify any patients who were RECIST-defined responders, which seemed to promote the detection sensitivity of responders. It seemed that α-RECIST increased the survival benefit of responders over non-responders compared with RECIST 1.1 [HR, 0.31 (95% CI: 0.10, 0.98); *p* = 0.046]. The α-RECIST criteria might be a promising tool for identifying tumor response of conversion-radiotherapy for uHCC before hepatectomy.

Pathologic response evaluation showed no significant correlation with neither RFS nor with α-RECIST evaluation. The pathologic response evaluation might only reflect the instant treatment response to radiotherapy, which may not translate into long-term survival benefit. 

Several limitations should be stressed regarding this study. First, the limited sample size was the main limitation. As a result, generalization of our conclusions should be interpreted with caution and prospective studies with larger sample size are urgently needed. Second, the primary endpoint was RFS after hepatectomy rather than OS in this study because OS may not reflect the survival benefit of IMRT and could be influenced by the subsequent therapies. Various therapies were utilized once recurrence occurred after initial hepatic resection (secondary resection, local ablation therapy, hepatic artery infusion chemotherapy, or systemic therapy).

## Conclusions

An optimal revised criterion (α-RECIST) was developed by combining the RECIST 1.1 with the AFP_Δ_ (cutoff value, 76%) to evaluate tumor response for uHCC receiving conversion-radiotherapy before hepatectomy. It was demonstrated that patients identified as responders by α-RECIST showed better RFS than those defined as non-responders. The α-RECIST criteria might be a promising tool for identifying tumor response of conversion-radiotherapy for uHCC before hepatectomy.

## Data Availability Statement

The datasets underlying this study are available on request to the corresponding authors. Requests to access these datasets should be directed to dr_fengye_ncc@163.com.

## Ethics Statement

This study involving human participants was reviewed and approved by Cancer Center/National Clinical Research Center for Cancer/Cancer Hospital, Chinese Academy of Medical Sciences and Peking Union Medical College, and was conducted in compliance with the 1975 Declaration of Helsinki, Good Clinical Practice guidelines and local regulatory requirements. Written informed consent was waived by the Institutional Review Board.

## Author Contributions

Study concept and design (YX, YY, and LL), acquisition of data (FY), analysis and interpretation of data (YX, YY, and LL), drafting of the manuscript (YX, YY, LL, and FY), critical revision of the manuscript for important intellectual content (FY), critical funding (MXZ and LL), administrative, technical, or material support, study supervision (FY and MXZ). All the authors have read and approved the manuscript and had access to the study data.

## Funding

This study was supported by the National Natural Science Foundation of China (No. 81971589) and the Youth Project of Beijing Hope Run Special Fund (No. LC2021B17).

## Conflict of Interest

The authors declare that the research was conducted in the absence of any commercial or financial relationships that could be construed as a potential conflict of interest.

## Publisher’s Note

All claims expressed in this article are solely those of the authors and do not necessarily represent those of their affiliated organizations, or those of the publisher, the editors and the reviewers. Any product that may be evaluated in this article, or claim that may be made by its manufacturer, is not guaranteed or endorsed by the publisher.

## Abbreviations

AFP, alpha fetoprotein; α-RECIST, alpha fetoprotein-RECIST; CE-MRI, contrast-enhanced magnetic resonance imaging; CR, complete response; CTV, clinical tumor volume; EASL, European Association for the Study of Liver Diseases; ECOG PS, Eastern Cooperative Oncology Group Performance Status; EPI, echo-planar imaging; HCC, hepatocellular carcinoma; HR, hazard ratio; IMRT, intensity-modulated radiotherapy; IQR, interquartile range; mRECIST, Modified Response Evaluation Criteria in Solid Tumors; OS, overall survival; PD, progressive disease; PFS, progression free survival; PR, partial response; PTV, planning target volume; RECIST 1.1, Response Evaluation Criteria in Solid Tumors, version 1.1; RFS, recurrence-free survival; RT, radiotherapy; SD, stable disease; SLD, sum of the longest diameter; uHCC, unresectable hepatocellular carcinoma; VEGF, vascular endothelial growth factor.
