# Supplementary material for: The α-RECIST (RECIST 1.1 Combined With Alpha Fetoprotein): A Novel Tool for Identifying Tumor Response of Conversion-Radiotherapy for Unresectable Hepatocellular Carcinoma Before Hepatectomy
Source: Front Oncol. 2022 May 24;12:905260. doi: 10.3389/fonc.2022.905260 (PMC9170883; doi:10.3389/fonc.2022.905260)
Supplement: Supplementary file 1 [file DataSheet_1.docx]

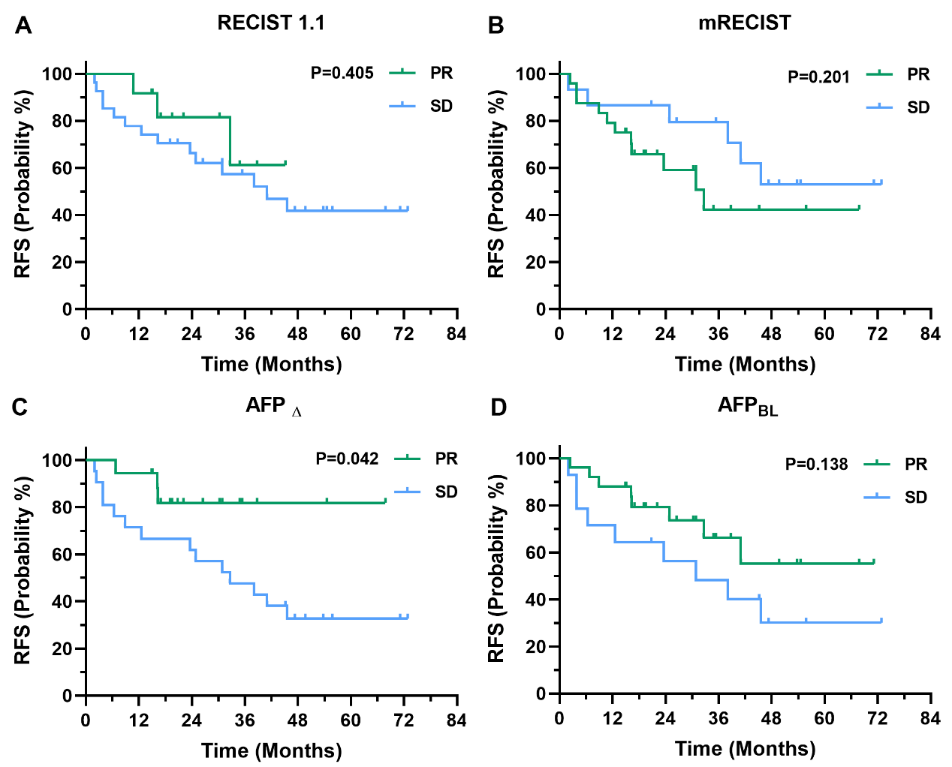


**Supplemental Figure 1.** Kaplan-Meier curves for RFS of 39 patients with uHCC undergoing IMRT before hepatectomy as categorized by the RECIST 1.1 (A), mRECIST (B) criteria, AFP_Δ_(C), and AFP_BL_ (D).


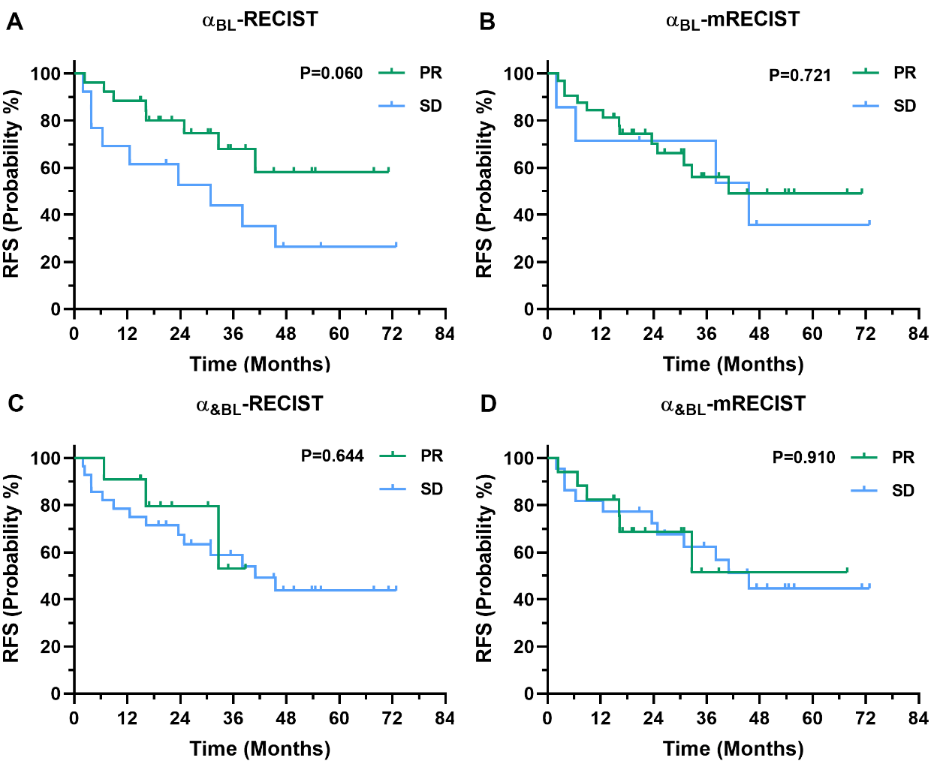


**Supplemental Figure 2.** Kaplan-Meier curves for RFS of 39 patients with uHCC undergoing IMRT before hepatectomy as categorized by the α_BL_-RECIST (A), α_BL_-mRECIST (B), α_&BL_-RECIST (C), and α_&BL_-mRECIST (D) criteria.


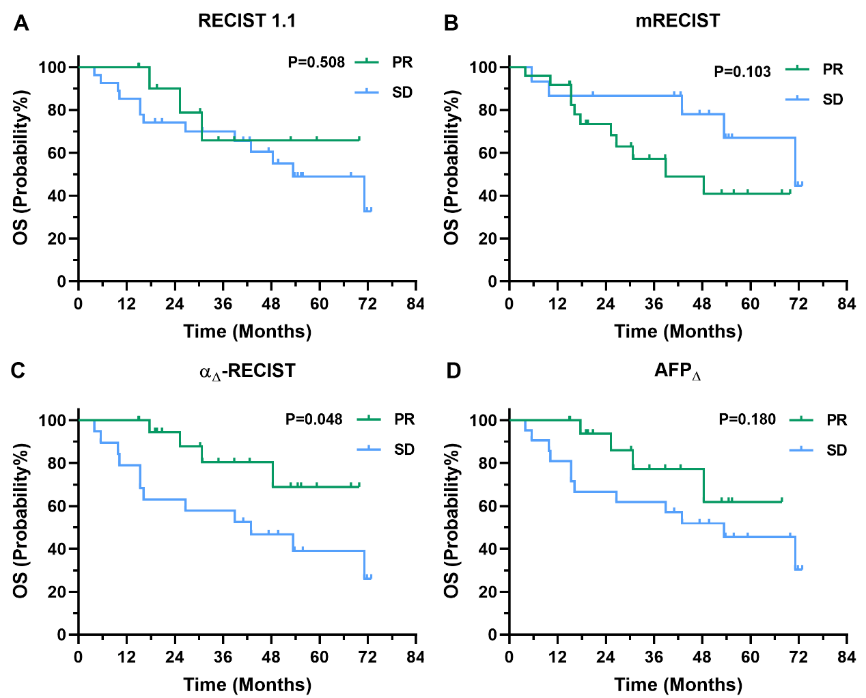


**Supplemental Figure 3.** Kaplan-Meier curves for OS of 39 patients with uHCC undergoing IMRT before hepatectomy as categorized by the RECIST 1.1 (A), mRECIST (B), α_Δ_-RECIST (C) criteria and AFP_Δ_(D).


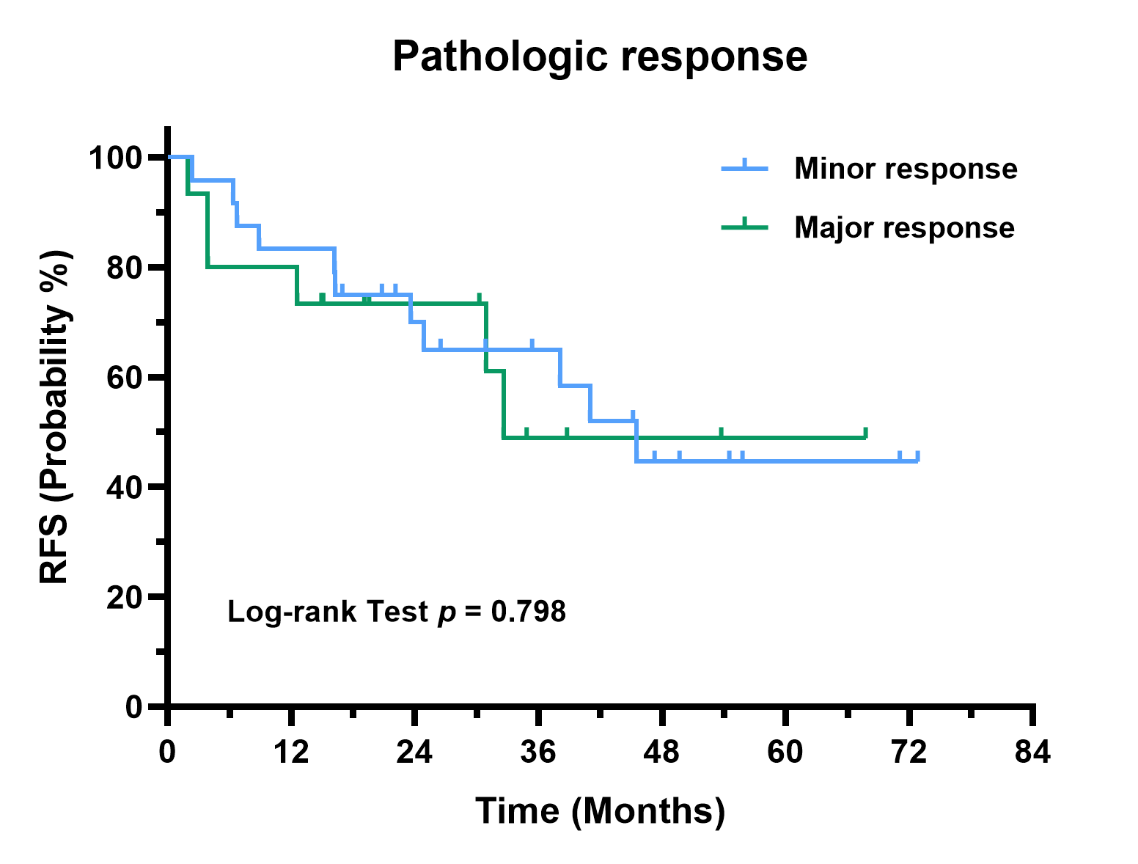


**Supplemental Figure 4.** Kaplan-Meier curves for RFS of 39 patients with uHCC undergoing IMRT before hepatectomy as categorized by the pathologic response.
